# Application of spherical substrate to observe bacterial motility machineries by Quick-Freeze-Replica Electron Microscopy

**DOI:** 10.1038/s41598-019-51283-w

**Published:** 2019-10-14

**Authors:** Eisaku Katayama, Yuhei O. Tahara, Clothilde Bertin, Satoshi Shibata

**Affiliations:** 1 The OCU Advanced Research Institute for Natural Science and Technology (OCARINA), Osaka City University, 3-3-138 Sugimoto, Sumiyoshi-ku, Osaka 558-8585 Japan; 2 Present Address: Waseda Research Institute for Science and Engineering, 3-4-1 Okubo, Shinjuku-ku, Tokyo 169-8555 Japan; 3 Present Address: Molecular Cryo-Electron Microscopy Unit, Okinawa Institute of Science and Technology Graduate University, 1919-1 Tancha, Onna-son, Kunigami-gun, Okinawa 904-0495 Japan

**Keywords:** Cellular microbiology, Cryoelectron microscopy

## Abstract

3-D Structural information is essential to elucidate the molecular mechanisms of various biological machineries. Quick-Freeze Deep-Etch-Replica Electron Microscopy is a unique technique to give very high-contrast surface profiles of extra- and intra-cellular apparatuses that bear numerous cellular functions. Though the global architecture of those machineries is primarily required to understand their functional features, it is difficult or even impossible to depict side- or highly-oblique views of the same targets by usual goniometry, inasmuch as the objects (e.g. motile microorganisms) are placed on conventional flat substrates. We introduced silica-beads as an alternative substrate to solve such crucial issue. Elongated *Flavobacterium* and globular *Mycoplasmas* cells glided regularly along the bead’s surface, similarly to those on a flat substrate. Quick-freeze replicas of those cells attached to the beads showed various views; side-, oblique- and frontal-views, enabling us to study not only global but potentially more detailed morphology of complicated architecture. Adhesion of the targets to the convex surface could give surplus merits to visualizing intriguing molecular assemblies within the cells, which is relevant to a variety of motility machinery of microorganisms.

## Introduction

Quick-freeze deep-etch-replica electron microscopy (hereafter QFDE) was, at first, established as a powerful technique to capture and visualize instantaneous structural features of solid tissues^1^ during rapid physiological events. Later, Heuser^2^ introduced mica-flakes as convenient adsorbents to immobilize small materials floating in solution and expanded the application so that the fine structure of macromolecular-assembly could be easily observed by the same technique^3^. Since quick-freeze replication has a great advantage over other means to give high-contrast details of individual protein-assemblies, it was effectively used to capture the structures involved in biochemical or biophysical experiments *in situ*, with the resolution high enough to enable us to discriminate even the conformational differences of single protein molecules during *in vitro* motility^4–6^. The same visualization method was also applied in microbiology to study the 3-D ultra-structure of various microorganisms such as *Chlamydomonas*^7^; *Trypanosoma*^8^; and yeast and fungi species^9^ or their body-parts, such as flagella^10^ and pili apparatuses^11,12^, involved in bacterial motility.

In those cases, target materials are usually placed on flat substrates as above or sometimes on a simple cover-slip, and instantaneously frozen by pressing onto a polished metal-block pre-cooled by liquid nitrogen, or more preferably by liquid helium^1^. The most detailed information on the surface profiles of the objects, is of course, obtained when the specimens are observed head-on from the top- the same direction as that of the rotation-axis of shadowing. Since replica specimens made of metal/carbon are quite robust to the electron-beam, it is easy to take a number of high-dose tilt-series micrographs of the target’s surface, some of which might be used to produce stereo-pairs or anaglyphs so that we can intuitively recognize the spatial arrangements of various architecture through our stereognostic sense^3,10,13,14^. Though we can readily recognize relatively simple surface profiles by stereo-views of tilted pairs, it is not necessarily easy to imagine side- or highly-tilted views of delicate 3-D profiles of complicated structures only from lightly-tilted views. This is not only because of the limitation of tilt-angles, but also by the halo obscuring the images of the objects floating apart from the background substrate (i.e. short-pitched helices of actin-filaments in Heuser & Kirschner^15^ or Morone^14^) or those projecting laterally from the main body (i.e. flagella in Katayama *et al*.^10^). We have been utilizing the QFDE method to investigate the structure of unconventional motility machinery of bacteria by a visualization of relevant protein assemblies *in situ*, especially under the most natural working states. In the case of gliding motility, the adhesion of the cells to solid surfaces requests dedicated proteins, generally called “adhesins“^16,17^ to invoke the motion. The molecular machinery responsible for such motility often includes a protein assembly that transmits the mechanical power developed by a set of intracellular engines toward the propelling apparatus on the cell surface, through a membranous boundary that separates the cytoplasm from the extra-cellular space. Especially for cells that adhere and glide along the surface of conventional flat substrates, it is extremely difficult or even impossible to observe the total appearance of the most intriguing “leg” portions of the microorganism under tension-bearing states, because they are located within the narrow interface between the substrate and the cell-body, largely covered by overriding cells^18^. A part of the molecular assembly within such space might be hidden, shaded by an overriding cell-body, and escape from the metal-vapor shadowing from substantially elevated angles. Thus, the use of conventional flat substrates poses severe restriction in achieving our goal. In order to obtain 3-D structural information on the architecture and/or arrangements of intra- and extra-cellular organelle, the most popular strategy might generally be electron tomography that integrates a number of tilted-views to give global 3-D image of the field^19,20^. Though the method is certainly powerful and sounds omnipotent for every kind of target, spatial resolution toward the depth in each reconstruction is actually limited because of the missing data-range problem that often causes severe ghosts along the Z-axis^21^. 3-D images of each target particle can be reconstructed only when low contrast images from a vast number of projections in various directions are collected and averaged. In order to overcome or at least minimize such a difficult issue, we developed a new algorithm that largely eliminates the ghosts from the tomogram^22^. Though its performance was particularly notable for high-contrast specimens like metal-replicas or silver-staining^23^, the results did not yet completely satisfy our final aim, and we looked for some original strategy to observe the real side-views of the targets. In the meantime, we had the opportunity to appreciate the value of real side-views of the targets under electron microscopy, which once more reminded us of the unparalleled advantage of direct side-view observation realized by somehow tilting the targets^24^. Thus, we started further attempts to achieve such a difficult, but indispensable goal by any means.

## Results and Discussion

### Selection of motile bacteria and their behavior on the substrates with non-flat surfaces

**Figure 1:**
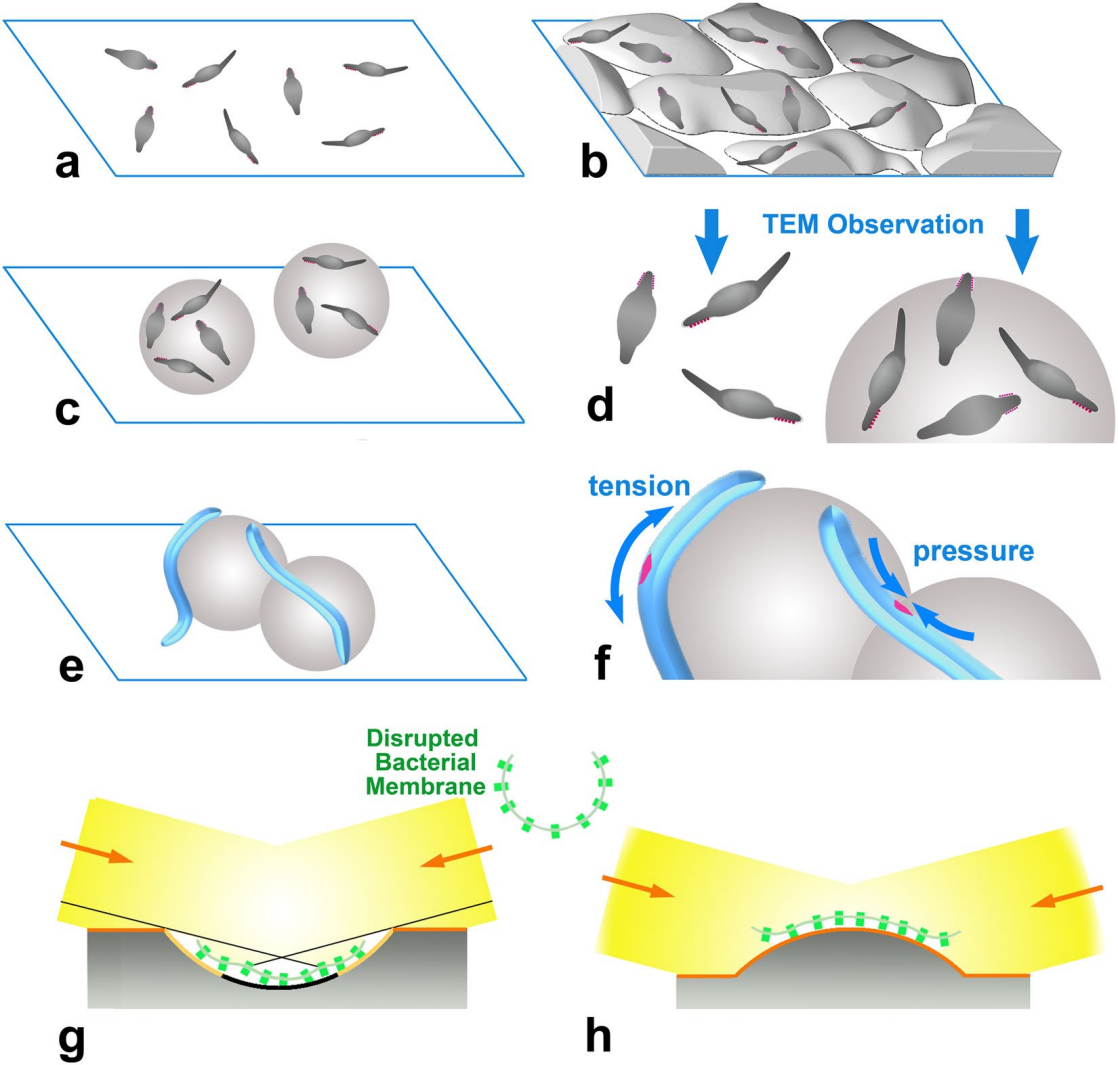
Schematic drawings to indicate the advantage of spherical beads as an alternative of conventional flat substrate for quick-freeze deep-etch replica electron microscopy. (**a**) Bacterial cells placed on flat surface give only the top- or slightly oblique-views by electron goniometry. (**b**) Though smoothed ground-glass did not successfully work for quick-freeze replica, it might be still useful as a substrate for the other surface observation apparatuses. (**c**) Spherical substrate provides the possibility of much wider view-angles including side-, oblique- and frontal-views of the targets. (**d**) Enlarged views of (**a**), left; and (**c**) right, respectively, with the direction to be observed in transmission electron-microscope. (**e**,**f**) Elongated bacteria attached along the spherical substrate also give various observation-angles (see the text for details). Attachment of those cells on the convex surface might forcefully extend the outer-side of the curved cell to facilitate weakening and regional opening of the cell wall. In the same way, bacteria cells that bridge two adjacent beads might receive substantial pressure at the elbow, partially exposing the internal structures. Pink-colored segments indicate the portions of internal structure revealed by imposed tension or the pressure as above. (**g**,**h**) Are the cartoons exhibiting two extreme cases for clarity, of rotary-shadowing (elevation-angle, 15°) to the targets along the inner-side of the bacterial membrane, where disrupted bacterial bodies (center) are strongly adsorbed to concave (**g**) and convex (**h**) substrates, respectively. In (**g**), protrusions along the inner membrane surface are either partly shadowed (light orange area) or not shadowed at all (black area), whereas all of the protrusions are evenly and fully-shadowed (orange area) in (**h**). Orange-colored-arrows indicate the direction of rotary-shadowing. Evenly metal-accumulated area increases, if the elevation-angle is changed during the procedure.

Actual attempts to work out our project started with a search for a new category of substrates with rugged surfaces instead of conventional flat-surfaces (Fig. 1a). At first, we tested ground-glass, after smoothing original sharp edges by treatment with hydrofluoric acid (Fig. 1b). Actually, the areas surrounding the peaks gave ample spaces with shallow slopes inclined up to 15 degrees, seemingly useful to observe substantially-oblique views of the adsorbed targets (data not shown), but we could not completely dissolve the thick glass substrate, even after two months.

As the next and more practical candidate, we explored the use of silica-beads (Fig. 1c), commercially available as various kinds of chromatography media. Considering the dimensions of the actual target cells, we chose beads with a 5 µm diameter.

Several bacterial species were selected as representative examples of elongated and spherical bacteria. The first elongated sample, *Flavobacterium johnsoniae* belongs to a family of the *Bacteroidetes* phylum and crawls up to 2 µm/s on several types of surfaces using cell surface adhesins SprB^25^ and RamA^26^. They are postulated to move along looped helical tracks operated by some motor proteins in the cell envelope^27^. The second, *Spiroplasma eriocheiris* is a Chinese mitten crab pathogen in the Mollicutes class, that can swim up to 5 µm/s by changing the helicity of its global shape at a kink traveling from the tip to the tail of the cell body^28^. The third and fourth, with more globular shapes, are two species of Mycoplasma genus. *M*. *mobile*, a fish pathogen, is known as the fastest gliding among its species. The latter, *M*. *pneumoniae*, a bipolar spindle-shaped bacterium is known as a clinically important pathogen that infects human lungs. They attach to sialic-acid components on the host cell surface with adhesin and spread along the surface of the cells that sometimes glides but very slowly^29^, with a short globular adhesin^30^. Since the gliding speed of the former is very rapid and easy to observe under optical microscopy, it was a good material to assess its interaction with a new substrate (details on its electron microscopic features will be published elsewhere).

**Figure 2:**
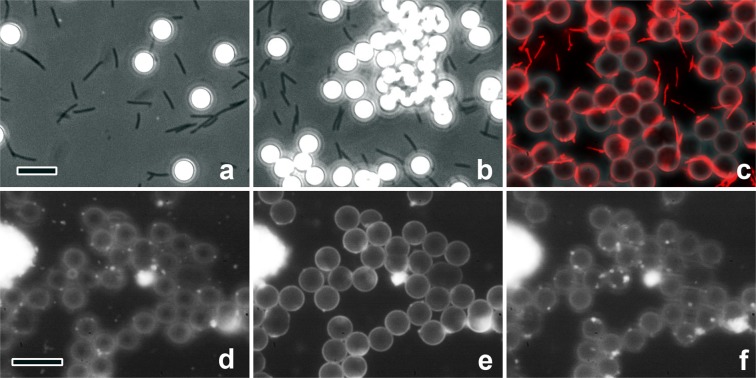
Sequential frames extracted from phase-microscopy movies, indicating the behavior of bacteria as reflected by bead movement. (**a**) Two minutes after addition of bacteria, spherical beads started to move with an increase of bacterial cells on the background. During the process, most beads eventually moved, for both species. (**b**) After more than 10 minutes, *F*. *johnsoniae* formed large aggregates of the beads, whereas *M*. *mobile* made only dual spheres (data not shown), reflecting the short cell dimensions. (**c**) Under fluorescence microscopy, live-stained elongated bacteria often attached to two beads simultaneously, forming bridges between beads, as observed in replica specimens. That might be the origin of large cluster-formation as above. (**d**–**f**) Images of globular *M*. *pneumoniae* with the beads in the same field, but at three different focal positions. (**d**) Focused at the level of background glass-surface, (**e**) at the level of bead’s center, (**f**) at the top of the beads. Scale-bars indicate 10 µm.

We first examined whether motile bacteria can closely interact and move regularly on such non-flat substrates. Since *F*. *johnsoniae* actively glides on non-coated glass, we simply mixed them with plain spherical beads free in solution and observed their behavior under phase-microscope (Fig. 2). Control silica-beads of this size stayed still at the original place, unaffected by Brownian motion. A few beads started to move slowly several minutes after addition of bacteria (Fig. 2a). Some elongated cells approached, hid behind the beads and left after a while. More and more fractions of beads moved according to the attachment of cells on the background, and finally formed large aggregates (Fig. 2b). Though the bacterial cell-bodies on the beads were hardly visible because of a much lower refractive index than that of the beads, such movement suggested that they might attach and crawl along the surface of the silica-beads, having a similar chemical property to the glass. Similar experiments were carried out with bacteria live-stained with fluorescent dye. If the microscopic focal levels changed, it was clear that elongated cell-bodies of the bacteria attached to the beads and crawled along its surface (Fig. 2c).

Since *M*. *mobile* is known as the fastest species among gliding bacteria, its motility must be easily observed under optical microscope. We checked its behavior to compare it with that of elongated bacteria. The cells mixed with silica-beads pre-coated with fetuin, a sialic-acid compound needed for the attachment of *Mycoplasmas*, then were put on a cover-glass and contiguously observed. The behavior of the beads was similar to that of *F*. *johnsoniae*, except that large aggregates were not formed, most probably reflecting much shorter cell-length. When the microscopic focus was properly adjusted, we could observe some cells slowly moving along the periphery of the beads (data not shown). Thus, *Mycoplasma* cells certainly attach and glide normally along the spherical surface in a similar manner to flat substrates. On the other hand, the same *Mycoplasma* cells mixed with poly-L-lysine-coated beads did not move, presumably because the cells are firmly and non-specifically attached to the beads. We also tested if *M*. *pneumoniae* regularly interacts with fetuin-coated beads in a similar manner to flat substrates. Since the attachment of the cells to free-floating beads was very weak, we pre-immobilized the beads briefly onto the cover-glass with highly-diluted collodion (less than 0.01%). To assess the attachment density of small cell-bodies along the uneven surface, they were live-stained with fluorescent dye and the pictures were taken at different focal levels (Fig. 2d–f). It was apparent that cells were bound to the bead’s surface, top or sides, with almost the same density as to the flat background.

### Electron microscopy of bacteria on spherical substrate

**Figure 3:**
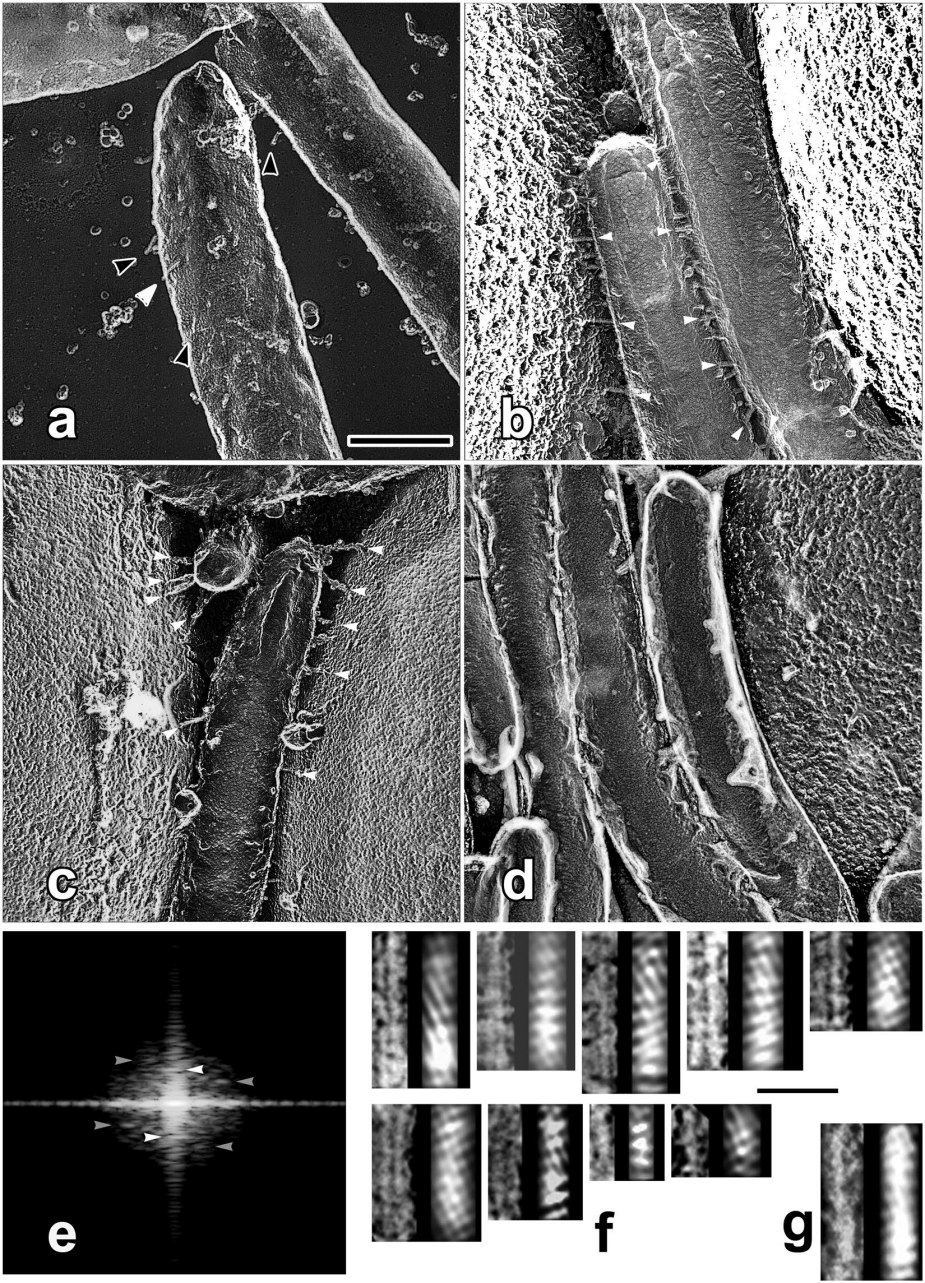
Quick-freeze deep-etch replica images of *F*. *johnsoniae* on flat (**a**) and spherical (**b**,**c**) substrates. (**a**) Leg-structures on the cell-surface were visible only on the top or on the background beside the cell-body (black and white arrowheads), whereas they were easily observed when spherical beads were used as the substrate (**b**,**c**). Note that the whole lengths of the legs protruding from the cell-body are clearly observed as side- or frontal-views in usual top-view (white arrowheads). Though any hint of specialized structures corresponding to the motor mechanisms was not observed around the root, legs grew denser and longer on spherical substrate. They could have been truncated on flat substrate. (**d**) Mutant cells that lack the relevant protein component (Δ*sprB*)^20^ did not show any leg structure. (**e**) Intensity distribution of accumulated FFT-power-spectra collected from FFT-diagram of nine “leg” segments (arrowheads in (**b**)). There are near-meridional (white arrowheads) and at least two off-meridional (gray arrowheads) layer-lines indicating the presence of helical molecular arrangements. Here, layer-lines are single-sided because only the top-surface of the helix is visible in rotary-shadowed replica specimens. Their axial repeat-lengths are 10.8, 17.0 and 8.5 nm, respectively. (**f**) Resultant series of filtered images of individual “leg” segments whose layer-line areas were selectively passed. Original (left) and filtered (right) images are paired side by side. Helical components in original images are clearly traced, suggesting the presence of such molecular arrangement in the “leg”-structure. (**g**) Control Fourier-filtered image of the “leg” segment on flat substrate (white arrowhead in (**a**)). Scale bars indicate 500 nm for (**a**–**d**), and 50 nm for (**f**,**g**), respectively.

We could regularly observe a number of bacterial cells with electron microscopy using conventional flat substrates. While the elongated shape of the *Flavobacterium* cell-body was clearly observed by QFDE on flat substrates (Fig. 3a), a hint of some leg structures (arrowheads) was sometimes seen at the top of, or on flat background beside the cell-body. However, the adhesins and motile apparatuses that actually contribute to the adherence or the motility of the cells are located along the bottom-side of the cell-body, and thus, it is apparently impossible to observe the total span of their structure under function.

Since we confirmed that both bacterial species attach regularly to spherical substrates, those on silica-beads were quickly-frozen as usual and subjected to deep-etch replication. Unlike insoluble ground-glass substrate, silica-beads were small enough to dissolve easily by hydrofluoric acid, and reproducibly provided good carbon-replica specimens, and not only frontal- and highly oblique-views, but also even complete side-views of many cells were readily observable without any tilting (Fig. 3b,c). Thus, the long-awaited side-views of the bacterial cells were effortlessly observed straight from the top, to give all the advantageous features of quick-freeze replica electron microscopy. Since the orientations of the cells were quite variable under this situation, metal-shadowing procedure segmented into dual- or multiple-angles (see Methods together with Fig. 1g,h) was highly effective to depict detailed surface-profiles of more evenly-shadowed, and much broader areas than those with conventional single-angle shadowing. Legs grew denser and longer on the spherical substrate, suggesting that they could have been somewhat truncated by the forced adsorption onto the flat substrate. Though the thickness of the legs was in the same range (ca 20 nm) as on a flat-substrate, the total span of the “leg” of motile cells was easily observed from the root to the tip under near-physiological conditions, promising us that more details on their structure or its changes during motility, if present, might be elucidated in the near-future (detailed results for *M*. *mobile* will be presented elsewhere). None of such leg structures were seen when mutant cells that lack SprB proteins were adsorbed to the beads (Fig. 3d). Filamentous legs of *F*. *johnsoniae*, known to consist of multimeric SprB components^25^, showed somewhat bumpy appearance in the replica images. Since bacterial bodies were suspended among the beads, legs were often straighter than on flat substrate, stretched by the natural tension, in convenience for quantitative structural analysis. Thus, their images were subjected to Fourier-analysis whether any hint of structural repeat might be present along their axes. Actually, accumulated FFT-spectrograms from many short segments indicated weak but solid signals corresponding to the presence of some helical structure with 10.8 nm as a main axial repeat (Fig. 3e–g). Unfortunately, however, we did not find any sign of specific motor-like structure at the root of the legs, suggesting that the propelling force by the legs might be generated somewhere in the periplasm or in intracellular space.

**Figure 4:**
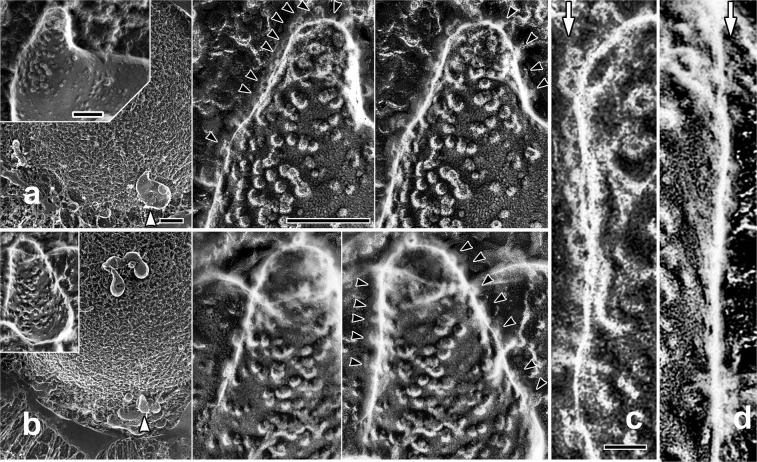
(**a**,**b**) Two sets of replica images of *M*. *pneumoniae* attached on silica beads. The cells located at the furthest deep-etched area from the center of the beads (indicated by white arrowheads) were observed. They are shown as enlarged views (upper-left insets). Further enlarged stereo-views (center) exhibit numerous stump-like structures on the back. Their diameter matched very nicely with that estimated by cryo-electron-tomography^30^. Side-views of the same structure (black arrowheads) are observed along the edges of the cell-body, some of which might be, at least, very close or potentially in contact with the substrate. Scale-bars for field-views (left panels) and enlarged-views (right panel) indicate 500 nm and 100 nm, respectively. (**c**) Further enlargement of the grazing view along the left edge of the cell in (**a**). Side- or deeply-oblique views of stump-like structures are clearly visible along the line indicated by a white arrow. (**d**) Similar grazing view of the control-cell which is less inclined against the view-angle. Stump-like structures might be barely visible along the periphery but far less conspicuously than in (**c**). Scale-bar exhibits 10 nm for (**c**,**d**).

Next, we made the attempt to visualize more difficult objects utilizing the new substrate. *M*. *pneumoniae* cells are thought to attach to the substrates through the stump-like structure called “nap”. Since the adhering complex has a globular structure with much shorter height as compared with the other adhesins^30^, it is challenging to observe its contact with the substrate within an extremely narrow space. For such purpose, we observed the cells that were attached at the furthest position from the center of the beads, where bacterial bodies should be substantially inclined to maximize visualization probability of the cell/substrate interface. Figure 4 indicates some of such areas. There, the cells in a typical *M*. *pneumoniae* shape exhibited a number of button-like protrusions on the back, which were condensed at the narrowed front-end, suggesting that the observed particles might certainly correspond to the “nap” structures reported to accumulate around such areas^30,31^. Seybert *et al*.^30^ conducted cryo-electron tomography of the whole *M*. *pneumoniae* cell and found the averaged diameter of the outer part of the surface protrusions to be 8 nm. Nakane *et al*.^31^, on the other hand, solubilized and isolated P1 adhesin/P-90 complex from the cell. They suggested that the nap structure might be a part of such complex. The approximate diameter of P1 adhesin/P-90 complex visualized by conventional rotary-shadowing was about 20 nm. The particles we observed along the back of the intact cells had a diameter somewhat less than 10 nm (Avg: 8.5 nm, S.D. = 2 nm, n = 127) and nicely matched with the result by tomography. Since their surface density seemed almost even along the cell-body’s circumference, the change of their shape according to the view-angles might reflect the different views of the nap. Emergence of non-circular side-views of the protrusions along the bottom area of the cell-body could possibly suggest a direct contact, or at least, a close approach of the nap to the substrate (see stereograms of Fig. 4a,b, together with Fig. 4c as compared with a control in Fig. 4d). Presence of a finer sub-structure might be suggested on some particles under high magnification. Though a minor fraction of smaller particles was present along the same surface, it is not yet clear whether different-sized particles consist of different combinations of protein-assembly.

### Further advantageous features available by spherical substrates

**Figure 5:**
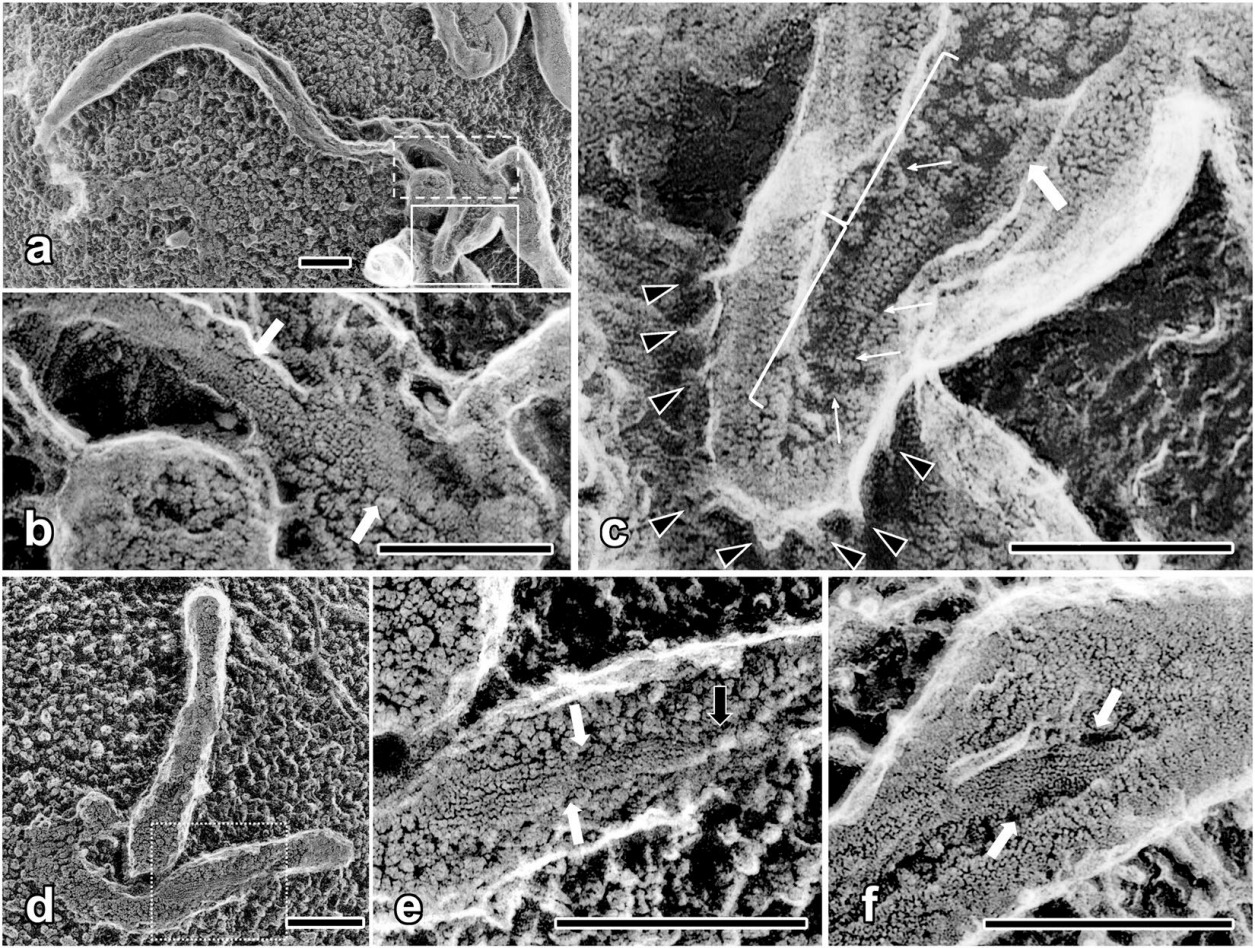
Replica images to exhibit characteristic features of intracellular structures within *S*. *eriocheris* cell *in situ*. (**a**) Low-magnification view of the entire *Spiroplasma* cell under its natural helicity, with a window opened on the cell membrane by an adsorption onto silica beads. Two kinds of internal structures^28^ related to cellular motility were exposed through two windows (indicated by dashed and continuous frames) which are exhibited as enlarged views in (**b**,**c**), respectively. (**b**) One of them is the bundles of intracellular filaments forming a characteristic fibril-ribbon that connects the shortest path (between thick white arrows) of the cell. (**c**) The other is a dumbbell-like structure (whose total span is shown by a bracket) bundled by fine rings (whose trajectory intermittently pointed by thin white arrows) at the tip pole of the cell^28^. Thick white arrow indicates the fibril-ribbon as above. Several cell surface spikes are visible along the tip extremity (black arrowheads). There could be some unknown scaffold supporting them from inside. (**d**) Another low-magnification view of a *S*. *eriocheris* cell absorbed onto a silica bead. The cell was broken into two and partly lost its helicity. Forced removal of the cell envelope at the tip pole of the cell exposed (**e**) the internal fibril-ribbon (two white arrows). The thickness of the ribbon reduced toward the cell extremity on the right, and its visible part ended in a “horse-tail node” structure (black arrow). Such node might prevent the assembled fibril-ribbon from unbundling into elementary filaments during the entire body’s twisting motion. (**f**) Close-up view of the fibril-ribbon (delimited by white arrows) at strongly-twisted portion of the cell, possibly during its natural motion. Since the materials were instantaneously fixed *in situ* by quick-freezing, such image might give a precious information on the biological structures under near-physiological functional states. Scale-bars exhibit 500 nm.

Surplus merits would be available by using such beads as a novel substrate. Most bacteria have a rigid cell-wall to survive under severe external stresses. Since their global curvature is preserved after physical disruption by osmotic shock, we had experienced considerable difficulty in the observation of molecular assemblies that are embedded along the hemi-spherical inner surface of the cell membrane^10^. On the other hand, the particles on the convex face should be more easily observed (Fig. 1g,h). It is noted that the targets along the inner-side of the membrane were best-visualized in inside-out membrane (compare Fig. 3 with Fig. 6c in Katayama *et al*.^10^, as an actual example). This is an intrinsic consequence of the metal-vapor shadowing emitted from the evaporation source at the top, and thus, the target cells tightly adhered to the spherical bead’s surface should have a tendency to form more convex faces to facilitate easier visibility. Further, the outer surface at the curvature of rod-shaped cells which were firmly adhered to the convex bead’s surface might receive substantial tension along their robust cell-walls. If cells under such conditions were subjected to osmotic-shock or other mechanical stress, the most extended portion at the outside elbow might receive the strongest tension that breaks up the local window through the envelope, and could expose a part of intracellular structures. Figure 5 exhibits one of such cases, using Spiroplasma species as a good example. *Spiroplasmas*, members of the Mollicutes, are long but very thin bacteria equipped with a membrane-associated fibril-ribbon to keep the cell’s helicity. They can swim in a liquid environment by twisting the whole body with a rapid motion of the same internal structure. Since they lack a cell-wall, their bodies are easily torn even with a weak osmotic shock. Figure 5 clearly indicates the effects of spherical substrates to the cell which is firmly adsorbed onto the silica-beads (Fig. 5a). Though its internal structures were described in the preceding literature^28^, we could observe even more detailed views of the same apparatuses in the cell membrane *in situ*, as 3-D replica images with higher contrast (Fig. 5b–e).

**Figure 6:**
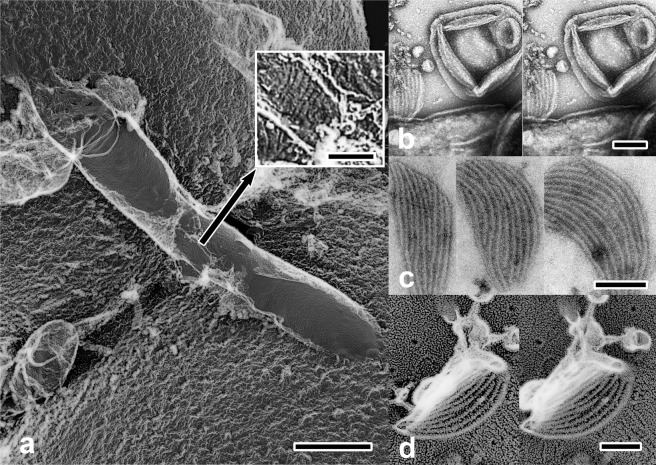
An example image to show partial opening of the window along the cell-wall of *F*. *johnsoniae*, by a forced adsorption to the beads. Scale-bar indicates 500 nm. Crystalline ladder-like structure (inset) was observed through the window at the flexed elbow of the cell. Right panels indicate (**b**,**c**) negatively-stained and (**d**) replica images of the membrane fractions prepared from the disrupted cells. Note that the intracellular lattice structure glimpsed through the opened window has white stripes of the width (~8 nm) and spacing (~15 nm) common to those observed in isolated membrane fractions, suggesting the identity of the structures. Extracellular adhesins are postulated to move along looped helical tracks, operated by some motor proteins in the cell envelope^27^. The observed structure could be a part of the architecture of that motility system^25,26^. Here, we simply show that our new method has a good potential to exhibit the presence of such intracellular-structures. More details will be published separately.

Long rod-shaped cells sometimes adhere tightly to two beads at the same time (Fig. 1e,f), as shown by fluorescent microscopy (Fig. 2c). Under such cases, the cell-body bridging adjacent beads might be sharply bent to make V-shaped distortion. Strong pressure imposed to the inner-side of the kinked portion permitted views of the cell envelope. In some favorable cases, we may be able to glimpse a part of intracellular apparatus (Fig. 6a) which, otherwise, is hard to work out by conventional handling. The grid-like appearance of the exposed apparatus might be a part of the intracellular helical track (Fig. 6b–d) along the surface of inner membrane that could support the motility of the bacteria^26^.

Thus, we have introduced a simple use and some applications of silica-beads as a new category of substrate for quick-freeze deep-etch-replica electron-microscopy. Target cells attached sideways to the spherical surface certainly provided the side- or highly-oblique views of various parts of the microorganism, which were difficult or almost impossible to achieve by any other means.

Such experimental setup, coupled with electron microscopy, might enable us not only to observe the global morphology of intra- or extra-cellular architecture but potentially to define their protein composition by a proper combination with antibody-labeling or avidin-biotin chemistry. For instance, if leg-portions of target cells are pre-immobilized tightly enough onto the beads (i.e. by brief chemical fixation), to keep their conjugation throughout the lengthy process, intracellular motor-assembly somehow connected to the extracellular legs might be exposed as described above and be visualized, with any luck, on the inner-side of the cell-membrane (see Fig. 5 of Katayama *et al*.^10^), still attached on the beads.

Since the novel experimental idea is quite simple in itself, it must be applicable in many ways, with a combination of a wide variety of other techniques. Though we employed specific beads that match to our purpose, beads with different properties are easily available. Such non-flat materials including ground-glass might be applicable as useful substrates for other surface visualization apparatuses such as atomic-force-microscopy^6^ or scanning electron microscopy. Application toward this line is in progress.

## Materials and Methods

### Bacterial culture

*S*. *eriocheiris* TDA-040725-5T and *M*. *pneumoniae* cells were cultured in R2 media at 30 °C^32^, or Aluotto media at 37 °C^33^, respectively. *F*. *johnsoniae* was grown in Casitone-yeast extract (CYE) medium. They were harvested and collected by centrifugation (Mycoplasmas and Spiroplasmas; 10,000 × g, Flavobacterium 5,000 × g at room temperature).

### Coating of the substrates for bacterial adhesion

Surface of the substrates for quick-freezing was sometimes coated with proper substances to facilitate the adhesion of bacterial cells.

Poly-L-lysine (Sigma-Aldrich) which is often used as an anchoring material of the eukaryotic cells to the glass was employed in some cases. Fetuin containing sialylated oligosaccharides is essential to keep the motility of *Mycoplasmas*. To avoid the bumpy appearance of original fetuin that could potentially hamper the visualization of small and delicate targets in QFDE images, we used protease-fragmented fetuin to coat the mica-surface. Fetuin (Sigma-Aldrich) (5 mg/mL) in 10 mM Tris-HCl (pH8) was subjected to Proteinase-K (Sigma-Aldrich) digestion at 1:5 ratio, for 30 min at 37 °C. The reaction was arrested by adding 5 mM phenylmethylsulfonyl fluoride, and the digest was passed through 10 kDa centrifugal filter; Ultracel-10 Centrifugal Filter-Unit (Sigma-Aldrich). The filtrate was used as the coating material of the substrates. Silica-beads with 5 µm diameter (Silica microspheres, Polysciences) were used after being processed in the same manner as flat ones.

### Optical microscopy of bacterial movement along silica-bead substrate

Bacteria were mixed with silica-beads and their movements were observed by phase-contrast microscopy. The motility of *F*. *johnsoniae* and *S*. *eriocheiris*, attached to the beads was video-recorded. In separate experiments, bacterial cells were live-stained with *N*-(3-Triethylammoniumpropyl)-4-(6-(4-(Diethylamino)phenyl)hexatrienyl)Pyridinium Dibromide (FM®4-64; Thermo-Fisher, T3166) and observed by fluorescence microscopy. In order to examine the attachment of total length of the elongated cells to the bead’s surface, pictures were taken at three different focal levels to make composite images. For that purpose, it was necessary to pre-immobilize the beads as follows. Highly diluted collodion (0.01% or less) in iso-amyl-acetate was put onto spread beads, immediately removed by a filter-paper and completely air-dried, before use, so that only the very base of the beads was glued to the flat bottom substrate. Video frames were processed with Image-J and Adobe Photoshop.

### Sample preparation for quick-freezing with mica-flakes and silica-beads

Mycoplasmas and Spiroplasmas were, at first, washed twice in phosphate-buffer (75 mM sodium phosphate; pH 7.3, and 68 mM NaCl) to remove the rich media components that could hamper the visualization. Then, bacterial samples were put on mica-flakes on aluminum freezing-discs and were incubated for 5 min. When silica beads were used as substrate, concentrated cells were put onto silica-beads which were mounted onto a thin layer of mica-flakes to separate them from rabbit-lung cushion. Since *M*. *pneumoniae* hardly attach to free-floating substrate, beads were pre-immobilized with highly diluted collodion as above, before use. After removal of excess liquid through the cushion, specimens were instantaneously frozen by a metal-contact with CryoPress (Valiant Instruments; St. Louis, MO) at liquid nitrogen temperature, or with QF-450 Quick-Freezing machine (Meiwa, Tokyo) at liquid helium temperature. The specimens were lightly knife-fractured, deep-etched at −104 °C for 4 min and were rotary-shadowed with platinum/carbon, followed by backing with pure carbon, basically according to Katayama^15^, but with JFDV Freeze-Etching Device (JEOL, Akishima, Japan). Though the evaporation-angle of shadowing was usually 17° (beads substrate) or 18° (mica substrate), dual-angle shadowing (e.g. 15° and 25° switched during shadowing) worked very effectively to cover the targets inclined to a variety of angles. Carbon-replicas were floated off by immersion onto the surface of hydrofluoric acid, cleaned with household bleach and picked up on 400-mesh copper or nickel grids. They were observed by JEM1010 transmission electron microscope (JEOL, Akishima, Japan) at 80 kV.

As a structural reference, the membrane fraction of the Flavobacterium periplasm was examined as follows. Cells were grown in 5 ml of motility medium (CYE 3-fold diluted to an optical density of around 1.0 at 600 nm) at 25 °C for 3 h and suspended in 100 µl of ice-cold sucrose solution (0.5 M sucrose, 0.15 M Tris-HCl, pH 7.5). After standing on ice for 15 min, the cells were mixed quickly with 1.4 ml of ice-cold water. Intact cells were removed by low speed centrifugation at 9,000 × g for 3 min. Osmotically shocked cells were collected from the supernatant by high-speed centrifugation at 20,000 × g for 5 min. The samples were stained negatively with 2% sodium-phosphotungstate (pH 7.0) or 0.5% uranyl acetate on a Butvar B-98 (Sigma-Aldrich) coated copper grid and were observed by transmission electron microscope.

### Image analysis of filamentous “leg” segments by fast-fourier-transform

Short straight segments were cut out from the replica images of the legs of *F*. *johnsoniae* attached onto silica-beads. They were subjected to Fast-Fourier-Transform by Digital-Micrograph (Gatan Inc.). FFT-spectrograms were accumulated to enhance weak signals from short segments. Then, layer-line areas were selectively passed for individual spectrograms to obtain filtered images of the originals.
